# Counting the Cost of Pins and Needles: A Case Study of Paresthesias and the Cost of Healthcare in the United States

**DOI:** 10.7759/cureus.59302

**Published:** 2024-04-29

**Authors:** Weilong Wang, Kayla Blackburn, Rebekah Lantz

**Affiliations:** 1 Internal Medicine, Wright State University, Dayton, USA; 2 College of Medicine, Boonshoft School of Medicine, Wright State University, Dayton, USA; 3 Internal Medicine, Miami Valley Hospital, Dayton, USA

**Keywords:** price transparency, test appropriateness, emr, encourage pcps, inflated healthcare spending, compunet, cost transparency, cost of healthcare, paresthesias, quality improvement (qi)

## Abstract

Healthcare costs in the United States (US) exceed those of comparable nations without yielding better outcomes. Factors contributing to this include lack of cost transparency, limited outpatient resources due to primary care provider shortages, and high patient volumes, where patients are not educated on differentials and the stepwise process of workup. Addressing these issues could curb unnecessary hospitalizations and expenses.

A 31-year-old woman with hypertension, alcohol use, anemia, and obesity experienced paresthesias in September 2022. At her first visit, the exam was consistent with decreased bilateral plantar sensation; however, there was no weakness or gait abnormality. This was not consistent with a focal neurologic distribution. Despite multiple ER visits, her condition persisted. Initial evaluations included potassium replacement ($80 for labs, $13 for tablet), nonacute head CT ($1500), and benign CT L-spine ($2500). Subsequent hospitalization led to brain MRI/MRA head/neck ($6700) and serum workup ($240), revealing deficiencies in vitamin D, folate, and B12. Treatment involved prednisone taper ($30) and supplemental vitamins ($35), with lifestyle recommendations ($0). After evaluating CompuNet lab costs and equivalent market imaging prices, potential savings exceeding $15,000 were identified through more focused and cost-conscious initial testing including vitamin studies and outpatient management, reducing hospitalizations and imaging expenses.

Rising healthcare costs in the US are driven by various factors, yet fail to correlate with improved outcomes. Our case argues that enhancing access to primary care, promoting cost transparency, and educating patients on healthcare decisions are crucial for mitigating excessive spending.

## Introduction

The United States (US) spends more on healthcare than other developed countries. According to data from 2020, the US spends 18.82% of its gross domestic product (GDP) on healthcare compared to 12.8% in Germany, 11.94% in the United Kingdom, and 11.8% in Switzerland [1]. Despite this, the US provides significantly fewer resources according to key measures of healthcare resources per capita including items such as hospital beds, physicians, and nursing staff [2]. More than 50% of excess spending is driven by a combination of factors such as higher administrative costs for physician insurance and insurance reimbursement, wages for nursing, midlevels, and physician provider staff, cost to stock and disburse prescriptions, as well as to afford appropriate level medical equipment [2].

Despite this higher cost of healthcare, the US does not consistently achieve superior health outcomes [3], emphasizing the need for a closer examination of healthcare practices and expenditures. In this context, addressing the issue of inappropriate medical testing is appropriate. Overtreatment contributes to waste in healthcare but also affects patients, ranging from financial burdens to patient anxiety and exposure to avoidable risks. Physicians play a pivotal role as stewards of healthcare resources, wielding clinical judgment, evidence-based practices, and effective communication to guide diagnostic decisions. As underscored by a study exploring overtreatment from physicians' perspective, the most common reasons include fear of malpractice, patient pressure or requests, and difficulty accessing medical records [4]. This reinforces the importance of refining medical practices to ensure that patient care aligns with best practices, fostering a balance between cost-effectiveness and optimal health outcomes.

Moreover, beyond the challenges posed by overtreatment, lack of price transparency in healthcare is of concern but a matter of complexity and is multifactorial. The price of medical services is set by public payers prospectively for providers and hospitals and is the result of negotiations between commercial health plans and providers [5]. The process of price setting creates many regulatory barriers to price transparency in healthcare.

Despite the barriers to price transparency, the benefits of price transparency drive the effort of its own expansion. A study reported that price transparency may yield between $17.6 and $80.7 billion in savings for patients, employers, and health plans by 2025 [6]. Benefits for patients include no surprise medical bills, ease to compare and choose providers, and more affordable healthcare services. For providers and hospitals, price transparency provides more upfront payment collections, and builds better patient trust and satisfaction [7].

In contrast to patients' expectations of receiving clear diagnoses and treatment plans, the complexities surrounding both overtreatment and the lack of price transparency in healthcare can significantly impact the fulfillment of these expectations. While patients anticipate a straightforward understanding of their medical conditions and corresponding treatment pathways, the prevalence of unnecessary medical tests due to factors like fear of malpractice or patient pressure can introduce ambiguity and potentially lead to unclear diagnoses [8]. Simultaneously, the lack of transparency in healthcare costs adds another layer of uncertainty for patients. When medical expenses are not readily disclosed, patients may find themselves navigating through a series of diagnostic procedures and treatments without a clear understanding of the financial implications [8]. This lack of clarity can create frustration and anxiety for patients who expect transparent communication about the costs associated with their healthcare.

To delve deeper into the practical implications of the healthcare issues discussed, we present a case involving paresthesias. Paresthesias refers to abnormal sensory symptoms often described as tingling, prickling, pins and needles, or burning sensations. They can be transient or persistent, affecting specific areas or the entire body connected to sensory or afferent nerve fibers. These sensations may occur on their own or be associated with reduced or absent feelings. Typically, the onset of paresthesias is acute [9]. Common diagnoses include vitamin deficiency, diabetic neuropathy, carpal tunnel syndrome, radiculopathy, multiple sclerosis, and peripheral vascular disease, most of which are reversible excluding diabetic neuropathy. Treatment depends on the origin of the pathology causing symptom onset. A study exploring patient satisfaction with the treatment of diabetic neuropathic pain revealed low satisfaction rates, with only 27% of patients reporting contentment with their treatment [10]. This finding underscores the challenges in addressing patient contentment in paresthesia care, where subjective experiences, persistent symptoms, and the multifaceted nature of treatment contribute to the complexities of achieving high satisfaction rates. By examining this case, we aim to provide a tangible context that underscores the real-world consequences of systemic challenges within the US healthcare system.

## Case presentation

In September 2022, a 31-year-old African-American woman with hypertension, alcohol abuse quantified as a fifth of vodka daily, anemia, and obesity (body mass index 30 mg/kg^2^) presented with bilateral plantar paresthesias, which started 1-1/2 weeks prior to presentation. She had a history of right ankle injury four years prior in 2018 while sliding down a hill, where management had been open reduction internal fixation of the right fibula but not had issues since. She had bilateral plantar neuropathy and intermittent numbness and tingling of the right upper extremity but there were no weakness or gait abnormalities. She had multiple healthcare visits in close succession.

During her first visit to the emergency room (ER), potassium was noted to be low at 3.1 mmol/L and repleted with 40 mEq/L oral potassium bicarbonate. Computed tomography (CT) head did not show an acute etiology and she was at low risk for stroke but instructed to follow up with outpatient neurology. She re-presented four days later to a different ER with similar neurologic complaints and constipation for three days. Additional laboratory tests included a basic metabolic panel, complete blood count with differential, and sedimentation rate, urine drug screen, pregnancy screen, urinalysis, thyroid function tests, coagulation labs, and liver profile. An X-ray abdomen showed mild constipation. CT cervical (C ) and lumbar (L) spine without contrast were negative for obstruction, stenosis, compression, or other lesions. Tests were within the normal range or noncontributory except for the elevated sedimentation rate as shown in Tables 1-2. She was prescribed prednisone 10 mg daily for 10 days for inflammation as well as famotidine 20 mg twice daily for two weeks for dyspepsia. She was advised to maintain her neurology appointment in a month and follow up with her primary care provider.

**Table 1:** Serum laboratory tests at the time of the second emergency room visit

Serum test	Value	Reference
CHEMICAL PANEL		
Sodium	136	136-145 mmol/L
Potassium	3.7	3.5-5.3 mmol/L
Chloride	102	98-107 mmol/L
Bicarbonate	25	21-31 mmol/L
Anion gap	9	7-16
Glucose	82	74-109 mg/dL
Blood urea nitrogen	12	7-25 mg/dL
Creatinine	0.7	0.6-1.1 mg/dL
Calcium	8.8	8.6-10.2 mg/dL
Magnesium	2.0	1.4-2.5 mg/dL
LIVER PANEL		
Total bilirubin	0.3	0.3-1.0 mg/dL
Alkaline phosphatase	55	34-104 U/L
Alanine transaminase	14	7-52 U/L
Aspartate aminotransferase	15	13-39 U/L
COMPLETE BLOOD COUNT		
Leukocytes	8.4	4.0-11.0 K/uL
Hemoglobin	12.0	12.0-16.0 g/dL
Platelets	345	135-450 K/uL
Mean Corpuscular Volume	89.2	80.0-100.0 fL
% neutrophils	61.9	%
THYROID STUDIES		
Thyroid-stimulating hormone	4.528	0.450-5.330 uIU/mL
Free thyroxine, T4	0.78	0.61-1.12 ng/dL
INFLAMMATORY MARKER		
Erythrocyte sedimentation rate	52	0-20 mm/hr
COAGULATION		
Prothrombin time	11.0	10.0-12.8 sec
Partial thromboplastin time	35.5	27.2-38.8 sec
International normalized ratio	1.0	0.9-1.1
ETHANOL	Not detected	Not detected

**Table 2:** Urine laboratory tests at the time of the second emergency room visit

Urine test	Result	Reference
URINALYSIS		
Color	Yellow	Straw/yellow
Clarity	Turbid	Clear
pH	6.5	5.0-8.0 pH units
Specific gravity	1.1025	1.001-1.035
Protein	Trace	Negative mg/dL
Glucose	Negative	Negative mg/dL
Blood	Trace	Negative
Nitrites	Negative	Negative
Leukocyte esterase	2	Negative
Pregnancy screen	Not detected	Not detected
DRUG SCREEN		
Amphetamines	Not detected	Not detected
Barbiturates	Not detected	Not detected
Benzodiazepine	Not detected	Not detected
Cocaine	Not detected	Not detected
Opiates	Not detected	Not detected
Marijuana	Not detected	Not detected

The patient visited a different ER for a third instance with the same complaint of plantar neuropathy without weakness. She was subsequently admitted due to recurrent visits. Vitals on admission were normal with 98.2℉, blood pressure 152/75, pulse 60 beats per minute, respirations 20 per minute, and oxygen saturation 100% on room air. Her exam was consistent with numbness to fine touch to the dorsum of bilateral feet. Her right lower extremity had more extensive sensory changes with numbness to the plantar and dorsal surfaces of the foot as well as to the ankle and leg without a specific dermatomal distribution. Magnetic resonance imaging (MRI) brain did not show white matter lesions disseminated in space in time and no regions of ischemia or hemorrhage and MR angiography (MRA) head and neck did not show vascular anomaly. During her hospitalization, general neurology was consulted. An autoimmune etiology or malabsorptive disorder was considered, so that testing was added including vitamins, heavy metals, autoimmune serologies, celiac panel, and serum and urine electrophoresis. Intracranial and spinal etiologies were determined to be less likely due to normal CT C-spine and L-spine, brain MRI, and head and neck MRA.

She was found to have borderline iron deficiency as well as folate and B12 deficiency. Further investigation into her diet entailed and found it to include mixed white and red meat, carbohydrates, sugars, and fats but limited in the fresh fruit and vegetable categories. The patient was discharged from the hospital with supplement prescriptions, daily 325 mg ferrous sulfate, folate 1 mg, cyanocobalamin 1000 mcg, and recommended for graduated alcohol cessation and healthful diet. Vitamin D-25,OH levels were pending at discharge but were found to be deficient ultimately (Tables 3-4).

**Table 3:** Autoimmune, vitamin, and mineral testing at the time of hospitalization ANA: antinuclear antibody; DNA: deoxyribonucleic acid; EIA: enzyme immunoassay; Ig: immunoglobulin; SSA: Sjogren's-syndrome-related antigen A autoantibodies; SSB: Sjogren's-syndrome-related antigen B autoantibodies

Serum test	Result	Reference
VITAMINS & MINERALS		
B6	8.1	2.1-21.7 ng/mL
B12	196	200-1100 pg/mL
D, 25-OH	17	30-100 ng/mL
Selenium	178	115-240 mcg/L
Copper	0.90	0.53-0.91 mg/dL
Zinc	12.56	9-14.7 mg/dL
Folate	4.7	>5.5 ng/ml
IRON STUDIES		
Iron	36	35-175 mcg/dL
Iron binding capacity	500	200-450 mcg/dL
Iron saturation	7	12-57%
Ferritin	10	12-156 ng/mL
AUTOIMMUNE TESTING		
Immunoglobulin A	266	81-463 mg/dL
Anti-endomysial IgA	Negative	Negative
Tissue transglutaminase IgA	<4.0	<4.0
Antigliadin IgA	<20	<20
Antigliadin IgG	<20	<20
Antinuclear antibody (ANA)	Positive	Negative
ANA pattern	Speckled	Negative
ANA titer	1:160	<1:40
Centromere IgG	<20	<20 EIA
Smooth muscle antibody	<20	<20 EIA
Ribonucleoprotein antibody	<20	<20 EIA
Sjögren's antibody (SSA)	<20	<20 EIA
Sjögren's antibody (SSB)	<20	<20 EIA
Scleroderma-70 antibody	<20	<20 EIA
Native DNA antibody	<20	<20 EIA
Histone antibody	<20	<20 EIA
Jo-1 antibody	<20	<20 EIA

**Table 4:** Electrophoresis results at the time of hospitalization A/G: albumin/globulin

Serum test	Result	Reference
HEMOGLOBIN ELECTROPHORESIS	No abnormal hemoglobin detected	No abnormal hemoglobin detected
Hemoglobin A	97.6	94.5-98.5%
Hemoglobin A2	2.4	2.0-3.3%
SERUM ELECTROPHORESIS	No M band present	No M band present
Total protein	6.1	6.0-8.3 g/dL
Albumin	3.2	3.5-5.2 g/dL
Alpha 1 globulin	0.3	0.2-0.5 g/dL
Alpha 2 globulin	0.8	0.5-1.1 g/dL
Beta globulin	0.9	0.6-1.1 g/dL
Gamma globulin	1.0	0.5-1.5 g/dL
A/G ratio	1.1	0.8-2.0

The patient had painful paresthesias in her right foot and suffered a motor vehicle accident the following month, which required operative repair with a transtibial screw. During her neurology appointment in October 2022, she reported mild improvement in paresthesias. Right upper extremity symptoms were more finitely described in an ulnar distribution so that local ulnar neuropathy was in question and electromyography was recommended, which ultimately showed normal amplitude, latency, and conduction volume for both motor and sensory studies. MRI C and T spine with and without contrast were devoid of demyelinating disease but showed mild disc bulges at C3/C4 through C6/C7 and varying foraminal narrowing C3/C4 through C5/C6. Small protruding posterior disc herniations were present at T7/T8 and T8/T9 as well as minimal left paracentral disc herniation at T10/T11. Images were not available within our system to provide here in this case report.

Compared to September levels, folate and B12 levels had improved to 309 pg/mL and 26 ng/mL, respectively. Vitamin D-25,OH levels had not improved (15 ng/mL) but apparently no supplementation had been started. Her iron levels were worse and vitamin D-25,OH were persistently low at follow-up but the patient endorsed continued alcohol use and poor compliance with supplements related to remembering to take them as well as desire. There were no cost issues for these supplements. She had constipation, which was a prior problem and a known side effect of iron tablets, so fiber and over-the-counter supplements were recommended. She preferred natural absorption of vitamin D from the sun but Fitzpatrick V skin tone was suspected to make this difficult and the patient endorsed often staying inside with a relatively sedentary lifestyle.

**Figure 1:**
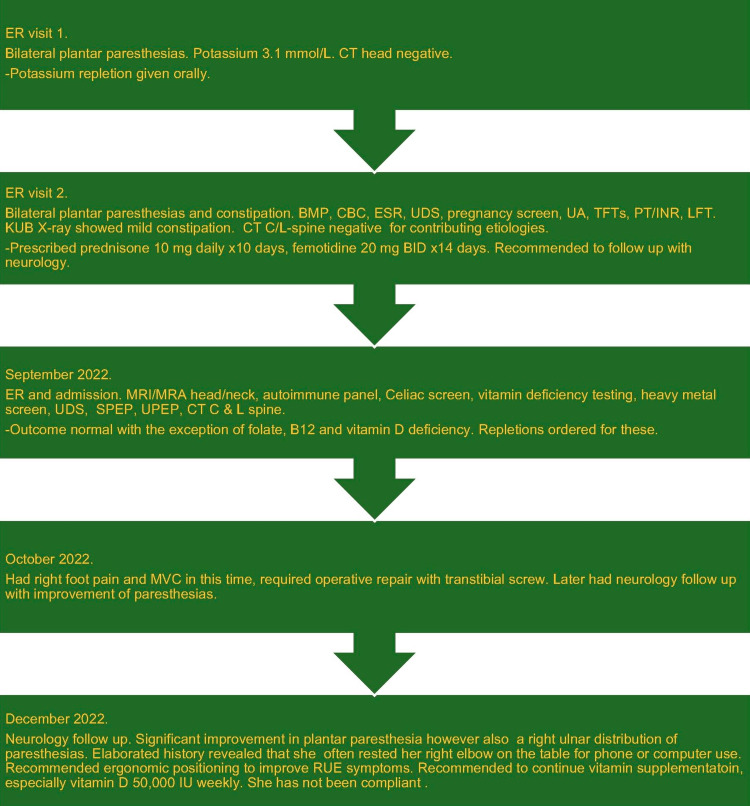
Timeline for the course of patient's paresthesias presentations BMP: basic metabolic panel; CBC: complete blood count; CT: computed tomography (C-cervical, L-lumbar); ER: emergency room; ESR: erythrocyte sedimentation rate; INR: international normalized ratio; KUB: kidneys-ureter-bladder; LFT: liver function test; MRA: magnetic resonance angiography; MRI: magnetic resonance imaging; MVC: motor vehicle collision; PT: prothrombin time; RUE: right upper extremity; SPEP: serum protein electrophoresis; TFTs: thyroid function tests; UA: urinalysis; UDS: urine drug screen; UPEP: urine protein electrophoresis

At her next neurology appointment, she reported significant improvement with paresthesia localized to an ulnar distribution, the last three fingers of her right hand. She would often rest her right elbow on the table to either use her phone or computer and she was recommended to limit this and given ergonomic advice given her right upper extremity symptomatology. She was recommended to continue vitamin supplementation, especially ergocalciferol 50,000 units weekly. She has not been compliant (Figure 1).

## Discussion

For the patient presented in the case, her medical costs are inflated related to multiple ER visits and progressive diagnostic studies for the same chief complaint. An important aspect of her care relates to cost analysis, which may elucidate why there are high healthcare costs in the US without an improved outcome.

Inpatient costs were not available despite many efforts to obtain them within-system and externally. The costs of diagnostic tests are discussed in terms of outpatient cash price instead in (Tables 5-6) using pricing provided by CompuNet Clinical Laboratories (CompuNet) [11] and imaging estimates from the Premier Mychart patient portal [12].

**Table 5:** CompuNet Lab test, ID, and comparative outpatient cost ANA: antinuclear antibody; BMP: basic metabolic panel; CAD: Canadian Currency; CBC: complete blood count; CMP: complete metabolic panel; ESR: erythrocyte sedimentation rate; HFP: hepatic function panel; HGB: hemoglobin; ID: identification; QUAL: qualitative; RA: rheumatoid arthritis; TIBC: total iron binding capacity; UA: urinalysis; UDS: urine drug screen; USD: United States Dollar Pricing provided by CompuNet [11]. Chart compiled by Rebekah Lantz.

Lab test common nomenclature	CompuNet ID number	Cost in USD
CBC	CBCD 300430, 500172	51.76
BMP	BMP 300066	33.84
CMP	CMP 300069	42.24
HFP	LIVR 300074	32.68
MAGNESIUM	MG 300301	26.80
PHOSPHORUS	PHOS 300344	18.96
B12/FOLATE	VB12P 300215, -237	111.12
IRON TIBC FERRITIN	FEPRF 300235, -285, -286	115.36
B6	VITB6 670881	112.40
B1	VITB1 670753	84.92
CHEMICAL UA	UMAC 300123	9.00
UA REFLEX TO CULTURE	UARFXM 300123	9.00
ESR	ESR 300488	10.80
QUAL 6-PANEL UDS	6UDSQ 700078	115.00
COPPER	COPPER 670226	49.64
SERUM SELENIUM	SELENS 670466, -8	102.12
SERUM SELENEUM	SELENR 670467	150.52
URINE SELENIUM	SELEU 670469, -470	181.38
ZINC	ZINCSP 670781	45.56
RETICULOCYTES	RETCCT 400432	15.96
HGB ELECTROPHORESIS	HGBEL 300262	51.48
PROTEIN ELECTROPHORESIS	SPE 300362	57.64
URINE ELECTROPHORESIS	UPE 300367	71.32
ANA & RA	ANARA 300501, -552	72.58
CELIAC PANEL	CELPK 500235, -240, -253, -254, -256	223.92

**Table 6:** Self-pay cost of imaging CT: computed tomography; MRA: magnetic resonance angiography; MRI: magnetic resonance imaging; USD: United States Dollar Pricing provided by Premier Mychartpatient portal [12].

Imaging study	Cost of test in USD
CT head without contrast	1038
Abdominal X-ray single-view	371
CT cervical and lumbar spine without contrast	2452
MRI brain with and without contrast	3259
MRA head and neck	3569
MRI cervical and thoracic spine without contrast	3254

The patient's initial presentation of dorsal pins and needles sensation with a subacute onset and no motor weakness, in the absence of trauma, is consistent with a diagnosis of sensory peripheral polyneuropathy [13]. A common cause would be diabetes mellitus, but the diagnosis was not present in her case and workup. Her alcohol use and malnutrition from poor diet led to multiple vitamin deficiencies. Her initial workup may have been approached as an outpatient rather than in the ER if she had presented stepwise and included vitamin deficiency labs, which would have been more cost-effective. If the supplement is not effective and the patient has compliance, more tests would then be warranted to explore further differentials. According to the cash price of outpatient tests from CompuNet [11], B12 and folate serum testing are $111.12 whereas involving CT, MRI, MRA, ER visits, and hospitalization raised the preliminary costs to >$15,000 USD [11,12].

This represents a single case in which the reality of healthcare expenditure in the US appears to be in excess compared to similarly developed status countries [14]. Table 7 provides a comprehensive comparison of all developed countries based on the Human Development Index (HDI) and their associated GDP [1]. HDI measures the average of human development based on knowledge, health longevity, and a decent standard of living apart from the economic growth of a country [1]. It is divided into four tiers: very high human development (0.8-1.0), high human development (0.7-0.79), medium human development (0.55-0.70), and low human development (<0.55) [1]. GDP is a measure of economic activity based on the value of goods and services, where a higher numerical value indicates a healthy economy. A physician-patient ratio (PPR) is important to consider, as the number may reflect an objective measure of access to care based on physician supply, and is also provided in the table. This does not designate primary care or specialist status of physicians and is physician-only, not accounting for the advanced practice providers who also serve our patients [14,15].

**Table 7:** Developed nations' HDI comparison and their associated GDP HDI: human development index; GDP: gross domestic product *Eurostat statistics [14], †WorldBank Indicator [15], “-” Indicates information not available. Chart compiled by Rebekah Lantz.

Developed country	HDI 2021	Latest Healthcare GDP (%)	Reference year	Physician-patient ratio †	Reference year
Switzerland	0.962	11.8 *	2020	4.4	2020
Norway	0.961	10.5 *	2020	5	2020
Iceland	0.952	9.6 *	2020	4.1	2019
Hong Kong	0.952	7 *	2021	1.3	1995
Australia	0.951	10.65 †	2020	4.1	2020
Denmark	0.948	10.5 *	2020	4.2	2018
Sweden	0.947	11.4 *	2020	4.4	2018
Ireland	0.945	7.1 *	2020	3.5	2020
Germany	0.942	12.8 *	2020	4.4	2020
Netherlands	0.941	11.1 *	2020	4.1	2020
Finland	0.94	9.6 *	2020	4.6	2018
Singapore	0.939	6.05 †	2020	2.5	2019
Belgium	0.937	10.9 *	2020	3.1	2019
New Zealand	0.937	10.03 †	2020	3.6	2020
Canada	0.936	11.68 †	2021	2.4	2019
Liechtenstein	0.935	6.5 *	2020	-	-
Luxembourg	0.93	5.8 *	2020	3	2017
United Kingdom	0.929	11.94 †	2021	3	2020
Japan	0.925	10.9 †	2020	2.5	2018
South Korea (ROK)	0.925	8.36 †	2020	-	-
United States	0.921	18.82 †	2020	2.6	2018
Israel	0.919	8.32 †	2020	3.6	2020
Slovenia	0.918	9.5 *	2020	-	-
Malta	0.918	9.2 *	2020	2.9	2015
Austria	0.916	11.5 *	2020	5.3	2020
United Arab Emirates	0.911	5.67 †	2020	2.6	2019
Spain	0.905	10.7 *	2020	4.4	2019
France	0.903	10.2 *	2020	3.3	2019
Cyprus	0.896	8.1 *	2020	3.1	2019
Italy	0.895	9.6 *	2020	3.9	2020
Estonia	0.89	7.8 *	2020	3.5	2019
Czechia	0.889	9.2 *	2020	4.2	2020
Greece	0.887	9.5 *	2020	6.3	2019
Poland	0.876	6.5 *	2020	2.4	2017
Saudi Arabia	0.875	5.54 †	2018	2.7	2020
Lithuania	0.875	7.5 *	2020	4.6	2019
Bahrain	0.875	4.72 †	2020	0.9	2015
Portugal	0.866	10.6 *	2020	5.5	2019
Latvia	0.863	7.5 *	2020	3.4	2020
Croatia	0.858	7.8 *	2020	3.5	2019
Andorra	0.858	9.05 †	2020	3.3	2015
Chile	0.855	9.10 †	2021	2.2	2019
Qatar	0.855	4.18 †	2020	2.5	2018
San Marino	0.853	8.69 †	2020	6.1	2014
Slovakia/Slovak Republic	0.848	7.2 *	2020	3.6	2019
Hungary	0.846	7.3 *	2020	3.5	2019
Argentina	0.842	9.98 †	2020	4.1	2020
Turkey	0.838	4.62 †	2020	1.9	2019
Montenegro	0.832	11.42 †	2020	2.7	2020
Kuwait	0.831	6.31 †	2020	2.3	2020
Russia	0.829	7.6 †	2020	3.8	2020
Brunei	0.829	2.39 †	2020	1.6	2017
Romania	0.821	6.3 *	2020	3	2017
Oman	0.816	5.33 †	2020	1.8	2020
Bahamas	0.812	7.59 †	2020	1.9	2017
Kazakhstan	0.811	3.79 †	2020	4.1	2020
Trinidad and Tobago	0.81	7.31 †	2020	4.5	2019
Costa Rica	0.809	7.68 †	2020	3.3	2020
Uruguay	0.809	9.15 †	2020	4.9	2017
Belarus	0.808	6.41 †	2020	4.5	2019
Panama	0.805	9.66 †	2020	1.6	2019
Malaysia	0.803	4.12 †	2020	2.3	2020
Serbia	0.802	8.73 †	2020	2.5	2015
Georgia	0.802	7.6 †	2020	5.1	2020
Mauritius	0.802	3.36 †	2020	-	-
Thailand	0.8	4.36 †	2020	0.9	2020

Potential solutions for improving healthcare costs may involve (1) recognizing appropriateness for ordering tests, (2) linking access across health records within an electronic medical record (EMR), (3) providing a steady supply of primary care physicians (PCP) and supplementary staff, and (4) improving price transparency.

Regarding appropriateness, Derakhshan et al. note that while biomedical sciences and technologies have improved in time, there is the risk of overutilization, overuse, and overtreatment, costing more than is medically useful, and contributes to at least 6-8% of total costs [16]. Clinically, this can lead to incidental findings with further unnecessary testing, over-prescription, over-imaging, and over-radiation. This can be physician-driven or patient-driven, as respective intrinsic and extrinsic factors [16]. Khattak et al. suggest asking a series of questions. Is the test logically consistent with the diagnostic hypothesis in question? Would any change in the clinical condition of the patient justify the test or its repetition? Can the test outcome change treatment decisions? How safe is the test? Is there any harmful consequence if the test is not ordered? [17]. For our patient, ideally, she would have presented first as an outpatient, and serum vitamin deficiency labs were ordered initially, given a nonfocal neurologic distribution of her symptoms. Her testing may have been as low as $300 or less compared to the >$15,000 that it was.

Record-sharing leads to improved patient outcomes. Much of charting has moved to EMRs; however, EMRs between systems frequently do not share data and can lead to repeat testing, oftentimes at high expense. Tu et al. discuss the benefit of a system that links records [18], which leads to improved comprehensive evaluation of a patient, their diagnostic workup, and outcomes of care.

Even more importantly, perhaps, is improving access to a steady supply of PCPs. A Veterans Affairs (VA) study involving a large sample of over five million patients between 2016 and 2019 assessed annual patient care costs [19]. The results were significant for a $3976 USD average saving based on descriptive statistics and multivariate regressions adjusted for severity of illness and confounders. The average total cost reduction for each subsequent PCP visit was $721 USD per patient per year. When patients were among the top 10% of high-risk patients, the first PCP visit involved a cost reduction of $16,406 USD, which accounted for 19% of the patient cost in that fiscal year [19]. PCP care absolutely contributes to reducing healthcare costs in society and improves patient outcomes. It would benefit national surveys to more broadly promote PCPs for population health.

Lastly, price transparency can benefit both the patient as an individual and on the national spending level. Prices are established by public payers on a prospective basis to physicians and hospitals. Commercial health plans then negotiate with healthcare businesses to determine charges and reimbursement [5]. Under the Centers for Medicare and Medicaid Services (CMS), there is little bargaining power on the part of businesses other than the election to not serve these patient populations. When patients as consumers are insured, there is a copayment or coinsurance amount while the insurance covers the remainder. While government institutions like the VA are not the direct drivers for cost, prices often correlate with Medicare fee-for-service as a marker within the market [5]. This is true for all services provided including lab-tests, pharmaceuticals, medical devices, provider pay, and hospital reimbursement. The Hospital Price Transparency rule went into effect on 1 January 2021, with studies such as Parente’s, estimating annual savings to consumers, employers, and insurers by 2025 [6]. The concept of this policy started during George W. Bush’s presidency (2001-2009) with the goal for Americans to note significant savings in their health expenses, having foreknowledge of price-of-care ahead of time, and the opportunity to choose among cost-effective options.

It took until 2019 during Donald J. Trump’s presidency to become an executive order for the federal regulation of visible healthcare prices for all consumers. The No Surprises Act became effective 1 January 2022 and provided that consumers enrolled in individual and group health insurance plans would be privy to knowledge of (1) hospital prices and (2) insurers’ negotiated prices with medical providers [6]. Despite these aims, as noted in our case study, cost is still not transparent so that providers are not aware, and charges are not disclosed openly to the patient. Fair price control through both state and federal regulations, focused on service, drug prices, and administrative costs, is needed to contribute to the solution.

There is likely much to learn from fellow developed countries as we present, such as those of comparable size, higher HDI, and lower GDP. Table 8 shows developed countries in order of decreasing population per the US Census Bureau [20], followed by HDI where a higher value is desired, the country’s GDP where a lower value is desired, and PPR where a lower number is desired. The top three comparators to the US with a population of 336 billion (B), HDI of 0.921, GDP 18.82 and PPR 2.6 were Russia (population 141B, HDI 0.829, GDP 7.6, PPR 3.8); Japan (population 123B, HDI 0.925, GDP 10.9, PPR 2.5); and Canada (population 39B, HDI 0.936, GDP 11.68, PPR 2.4) [1,15,20].

**Table 8:** Comparators to the United States for Healthcare Measures HDI: human development index; GDP: gross domestic product *US Census Bureau [20], †WorldBank Indicator [15]. Chart compiled by Rebekah Lantz.

Developed country	Country population 2024*	HDI 2021	Latest healthcare GDP (%)	Reference year	Physician-patient ratio †	Reference year
United States	336,673,595	0.921	18.82 †	2020	2.6	2018
Russia	140,820,810	0.829	7.6 †	2020	3.8	2020
Japan	123,201,945	0.925	10.9 †	2020	2.5	2018
Canada	38,794,813	0.936	11.68 †	2021	2.4	2019

Our case elucidates an area of improvement within US healthcare, which is the cost of healthcare. While spending more, outcomes are not shown to be improved compared to similarly developed countries. The closest comparators are Russia, Japan, and Canada by population size, HDI, GDP, and PPR, with different structures and systems of care. We note solutions for improving healthcare costs would likely involve ordering appropriateness and stewardship, linking EMR, outputting PCPs, and adherence to cost transparency regulations.

## Conclusions

Based on the literature and our case report, we show how the US overspends in healthcare without improved clinical outcomes compared to similarly developed nations of the nearest size, Russia, Japan, and Canada. US spending by GDP is higher (18.82%) than that of Germany (11.94%), the UK (11.94%), and Switzerland (11.8%). Overtreatment contributes to resource waste and imposes a financial burden on patients. On the end of ordering providers, this appears to be driven by fear of malpractice, patient pressure, and difficulty accessing unlinked medical records. Price transparency was thought of as an achievable solution and was addressed by the No Surprises Act in 2019 but does not appear to have been implemented in practice. Costs still remain vague, and charges are not openly disclosed to ordering providers or to patients.

The patient in this case presented with paresthesias without a neurologic distribution, which may have ideally been addressed in the outpatient setting starting with vitamin deficiency testing. At that time with clear history taking, her poor diet and alcohol abuse may have been uncovered with actions toward lifestyle changes. She presented to the ER instead of to a PCP, affecting downstream care and costs. In healthcare, we emphasize a balance between providing good clinical care and cost stewardship, using a paresthesia presentation example. We express solutions including training in appropriateness criteria, linking EMRs, turning out a steady supply of primary care providers, and cost transparency.
